# Exogenous Melatonin Alleviates NaCl-Induced Salinity Stress in Forage Pea (*Pisum sativum* L.): Concentration Optimization and Genotype-Specific Responses

**DOI:** 10.3390/metabo16060407

**Published:** 2026-06-10

**Authors:** Melih Okcu, Zuhal Okcu, Funda Kaya, Kamil Haliloglu

**Affiliations:** 1 Department of Field Crops, Faculty of Agriculture, Ataturk University, Erzurum 25240, Türkiye; 2 Department of Gastronomy and Culinary Arts, Faculty of Tourism, Ataturk University, Erzurum 25240, Türkiye; 3 Department of Biology, Faculty of Science, Gazi University, Ankara 06500, Türkiye

**Keywords:** foliar biostimulant, oxidative stress mitigation, cool-season legume, phenotypic plasticity, antioxidant defense, sustainable forage production

## Abstract

**Highlights:**

**What are the main findings?**

- Across 13 forage pea genotypes, 100 µM exogenous melatonin alleviated moderate NaCl-induced stress, lowering canopy temperature by ~4.4 °C and restoring SPAD values to near-control levels under 200 mM NaCl, whereas supra-optimal doses (150–200 µM) produced a paradoxical phytotoxic response under severe salinity.
- Emirbey and Kirazlí sustained the highest vegetative growth under salinity, while Özkaynak retained the greatest chlorophyll content, identifying complementary breeding targets for salt-affected environments.

**What are the implications of the main findings?**

- Pairing a 100 µM foliar melatonin application with targeted genotype selection (Emirbey/Kirazlí for biomass, Özkaynak for chlorophyll stability) provides a tractable, low-input strategy for forage pea on moderately saline soils.

**Abstract:**

**Background/Objectives**: Soil salinity is a major constraint on legume productivity worldwide, threatening forage pea (*Pisum sativum* L.) cultivation in semiarid regions. This study evaluated the effect of exogenous melatonin in attenuating NaCl-induced salinity stress across diverse forage pea genotypes. **Methods**: A three-factor factorial experiment was conducted under greenhouse conditions, testing three NaCl levels (0, 100 and 200 mM) and four melatonin concentrations (0, 100, 150 and 200 µM) across 13 genotypes with three replications (468 pots). Nine vegetative traits were measured and analyzed by factorial ANOVA and Tukey’s HSD test. **Results**: Increasing NaCl from 0 to 200 mM reduced plant height by ~28% and node number by ~32%. Application of 100 µM melatonin under 100 mM NaCl reduced canopy temperature from 28.1 °C to 23.7 °C and restored SPAD values from 21.7 to 26.5 under 200 mM NaCl. By contrast, 200 µM melatonin under severe salinity paradoxically suppressed SPAD to 8.9 and reduced root length. Emirbey and Kirazlí showed the greatest vegetative growth, while Özkaynak exhibited the highest chlorophyll content. **Conclusions**: 100 µM melatonin emerged as the optimal concentration for alleviating moderate salt stress in forage pea, and genotype selection is critical when deploying melatonin as a biostimulant under saline conditions. Direct measurement of biomass, yield, and forage quality under field conditions remains an essential next step before agronomic deployment.

## 1. Introduction

Soil salinity is one of the most pervasive and destructive abiotic stresses confronting global agriculture. The most recent FAO assessment estimates that approximately 1.38 billion hectares of land—10.7% of the global land area—are already affected by salinity, with a further billion hectares at risk under the combined pressures of climate change and unsustainable land management [1]. An estimated 1.5 million hectares of farmland are lost annually to salt accumulation, and modelling of global aridity trends suggests that the affected surface could expand to between 24% and 32% of total land under current temperature trajectories [1,2]. In Türkiye, approximately 1.52 million hectares of agricultural land (~5.5% of cultivable area) are salt-affected, with particularly severe conditions documented in the Harran Plain and the Great Konya Basin, where secondary salinisation driven by improper irrigation continues to expand the affected area [2,3,4].

Salinity affects plants through two coupled mechanisms: an initial osmotic phase in which excess NaCl lowers soil water potential, restricting water uptake and inhibiting leaf expansion [5]; and a subsequent ionic phase characterised by toxic accumulation of Na^+^ and Cl^−^ in plant tissues, with consequent disruption of enzyme function, displacement of K^+^, and generation of reactive oxygen species (ROS) that oxidise proteins, lipids and nucleic acids [6,7]. Legumes, being glycophytic, are particularly vulnerable: growth, nitrogen fixation, and nodule formation are appreciably compromised even at moderate NaCl concentrations (50–100 mM) [2,8]. Comparable patterns of salt-induced impairment have been documented in cowpea and other traditional grain legumes, where germination, photosynthesis, and seed yield decline progressively above 50–75 mM NaCl [9,10].

Forage pea (*Pisum sativum* L. var. *arvense*) is a cool-season annual legume of considerable importance in temperate and semiarid cropping systems, providing high-protein biomass (typically 18–28% crude protein on a dry-matter basis) and contributing to soil fertility through biological nitrogen fixation [11,12,13]. The species is, however, classified as moderately salt-sensitive: germination rate, seedling vigour and relative water content decline progressively above ~50 mM NaCl, and yield losses approaching 60–70% have been reported at 150–200 mM NaCl in greenhouse and field assays [4,14]. Despite this sensitivity, no commercial salt-tolerant forage pea cultivar is currently available, and conventional breeding for salinity tolerance is constrained by the relatively narrow genetic variation for salt-responsive traits in commercial germplasm and by the polygenic, environment-dependent nature of the trait [14,15]. Agronomic management strategies—leaching, gypsum amendment, drainage—remain costly and only partially effective in salt-affected smallholder systems. These limitations have prompted increasing interest in chemical priming and biostimulant approaches as practical, field-deployable means of mitigating salt stress in pea, yet validated chemical interventions for *P. sativum* under NaCl stress remain scarce.

Among emerging biostimulants, melatonin (N-acetyl-5-methoxytryptamine) has attracted substantial attention. First identified in plants in 1995, it is now recognised as a multifunctional indoleamine that acts both as a direct radical scavenger and as an inducer of the enzymatic antioxidant system, upregulating superoxide dismutase, catalase, peroxidase and ascorbate peroxidase activities under stress [16,17]. Sharing tryptophan as a precursor with indole-3-acetic acid, melatonin also modulates auxin-mediated growth and influences expression of salt-responsive genes (SOS, NHX, DREB), promoting Na^+^ exclusion and K^+^ retention—mechanisms central to ionic homeostasis under salt stress [18,19,20]. A recent meta-analysis encompassing more than 60 studies identified 50–200 µM as the broadly effective concentration range, while emphasising that efficacy varies markedly with species, application method, and stress severity [21].

Despite these advances, the response of *Pisum sativum* to exogenous melatonin under NaCl stress remains poorly characterized. Existing work in pea has focused largely on drought rather than salinity [22], and the few salinity studies have examined one or two genotypes, leaving the extent of genotypic variation in melatonin responsiveness essentially unexplored. Given the substantial genetic diversity within forage pea germplasm for abiotic-stress traits [23,24], a multi-genotype factorial assessment is required to identify both the optimal melatonin concentration and the genotypes most amenable to biostimulant-mediated alleviation of salinity stress. The recent literature has illuminated key mechanisms by which salt stress disrupts cell expansion and growth: the reorganisation of cortical microtubules and interphase microtubule arrays [23,24], alterations in the accumulation of osmoprotectants such as proline, betaine and soluble sugars [25,26], and the triggering of programmed cell death pathways under severe ionic stress [27,28]. Melatonin’s effect on growth under salinity may be mediated through modulation of these cytoskeletal and metabolic responses, but the mechanistic pathways remain incompletely characterized in legumes.

The novelty of the present work resides in three converging contributions that distinguish it from prior melatonin–salinity studies. First, while melatonin’s amelioration of salt stress has been documented in cereals, horsegram, common bean, and alfalfa, comparable evidence for forage pea (*Pisum sativum* var. *arvense*) remains limited to single-genotype experiments [22], leaving the role of genotypic variation entirely unaddressed. Second, the simultaneous evaluation of 13 widely cultivated genotypes is justified on physiological grounds: forage pea displays well-documented variation in root architecture, leaf-water relations, and chlorophyll fluorescence under abiotic stress [23,24], and these traits in turn determine the magnitude of biostimulant response. Restricting investigation to one or two genotypes—as in most published studies—forecloses identification of the genetic backgrounds most responsive to biostimulant intervention, an essential prerequisite for translating laboratory findings into breeding programs. Third, the three-factor factorial design (G × S × M) systematically resolves the joint contribution of genetic, environmental, and chemical factors, yielding interaction terms that single-factor or two-factor studies cannot detect. To our knowledge, this represents the largest published genotype × salinity × melatonin matrix in any legume crop.

Accordingly, the present study was designed to assess the effect of exogenous melatonin concentration and genotypic background on the response of forage pea to NaCl-induced salinity stress, with the dual objective of identifying the optimal melatonin dose for stress alleviation and characterising genotype-specific melatonin responsiveness across 13 cultivated genotypes.

## 2. Materials and Methods

### 2.1. Experimental Location and Plant Material

The experiment was conducted in 2023 in the greenhouses of the Plant Production Application and Research Center at Atatürk University (Erzurum, Türkiye; 39°54′ N, 41°16′ E; altitude 1869 m a.s.l.). Thirteen forage pea (*Pisum sativum* L.) genotypes widely cultivated in various regions of Türkiye were used as plant material: Tore, Taşkent, Emirbey, Ürünlü, Paslı, Gölbaşı, Bölükbaşı, Ovacevirme, Sayvan, Karptalınar, Kirazlı, Özkaynak, and Öncül. These genotypes represent the broad genetic diversity present in Turkish forage pea germplasm and were selected to capture a wide range of potential responses to abiotic stress.

### 2.2. Pot Preparation and Growing Conditions

The experiment was conducted in 5-litre plastic pots, each filled with 4500 g of a growing medium composed of field soil, peat, and coarse sand (2 mm particle size) in a 1:2:1 (*v*/*v*/*v*) ratio. All pots were slowly saturated with water from the base until drainage appeared through the drainage holes, and were subsequently covered with aluminium foil for 48 h to allow equilibration. After the equilibration period, each pot was weighed individually to determine the field capacity moisture content. Sowing was performed immediately thereafter, with four seeds placed per pot at a uniform depth.

At sowing, chemical fertilizers were applied at rates of 16 kg da^−1^ ammonium sulphate [(NH_4_)_2_SO_4_] as the nitrogen source and 18 kg da^−1^ triple superphosphate (TSP) as the phosphorus source, according to standard forage legume production practices. Plants were grown under controlled greenhouse conditions at 20 ± 1 °C with a 16 h photoperiod provided by Philips TL-D 36W/840 cool-white fluorescent lamps supplemented with natural daylight through glass panels, with a combined photosynthetic photon flux density (PPFD) of 300 µmol m^−2^ s^−1^, measured with a quantum meter (LI-COR LI-250A, Lincoln, NE, USA) at midday. An 8 h dark period was maintained for the first 15 days following sowing.

### 2.3. Experimental Design and Treatments

The experiment was arranged as a three-factor factorial in a randomised complete block design with three replications. The three factors were: (i) genotype (G), comprising 13 forage pea genotypes; (ii) salinity (S), comprising three NaCl levels (0, 100, 200 mM); and (iii) exogenous melatonin (M), comprising four concentrations (0, 100, 150, 200 µM). The total number of experimental units was 13 × 3 × 4 × 3 = 468 pots.

The four melatonin concentrations (0, 100, 150, 200 µM) were selected on the basis of the meta-analysis of Qi et al. [21], which identified 50–200 µM as the broadly effective range for salt-stress alleviation across plant species, and on prior studies in legumes specifically (effective range 50–250 µM) [22,29,30]. Concentrations below 100 µM were not included because preliminary work in our laboratory indicated negligible effects of 50 µM melatonin on forage pea seedlings under saline conditions; the supra-optimal doses (150, 200 µM) were retained specifically to test the existence of a phytotoxicity threshold.

NaCl treatments were initiated 15 days after sowing using analytical-grade NaCl dissolved in tap water. Irrigation was applied every three days at field capacity (five irrigations per pot during the 15-day treatment period); each event restored the pot weight to field capacity using the respective salt solution, ensuring uniform and sustained osmotic stress.

Melatonin solutions were applied as foliar sprays using a handheld pneumatic pump sprayer (0.5 L capacity, adjustable nozzle) to ensure uniform coverage of the leaf surface, with each spray application delivering exactly 5 mL per pot as measured by volumetric syringe to standardize the dose. All spray applications were conducted in the early morning (08:00–09:00 h) to minimize leaf surface evaporation and promote absorption. Melatonin (N-acetyl-5-methoxytryptamine; Sigma-Aldrich, ≥98% purity, M5250) stock solutions were prepared in a small volume of ethanol and diluted to the target concentrations with distilled water. Foliar applications commenced on the day of NaCl initiation and were repeated every two days for a total of seven applications, with each event ensuring complete coverage of the leaf surface. Multiple spray applications were performed to replicate standard foliar nutrition and biostimulant practices in commercial forage production, where split applications improve bioavailability and reduce risk of phytotoxicity; the two-day interval was chosen based on the demonstrated persistence of melatonin effects in previous legume studies [31,32]. The 0 µM control received an equivalent volume of distilled water containing the same ethanol carrier concentration. Plants were harvested 15 days after the commencement of treatments, at which point all measurements were recorded.

### 2.4. Traits Measured

At harvest, nine vegetative traits were recorded on a per-plant basis. Plant height (cm) was measured from the soil surface to the apical growing point of the main stem using a ruler to the nearest 0.1 cm. Root length (cm) was determined after careful removal of the root system from the pot and washing under running water, by measuring the primary root from the root–shoot junction to the tip with the same precision [33]. The number of nodes per plant was determined by counting all leaf-bearing nodes on the main stem. Leaf width and leaf length (cm) were each calculated as the mean of three measurements taken on the middle leaflet at the 4th, 5th and 6th nodes from the base using a digital calliper (precision 0.01 cm). Internode distance (cm) was measured between the 4th and 5th nodes on the main stem using a ruler. The number of main branches was recorded as the mean count of primary lateral branches originating from the main stem across five plants per experimental unit. SPAD value (relative chlorophyll content) was measured non-destructively on the most recently fully expanded leaves of five plants per pot using a SPAD-502 chlorophyll meter (Konica Minolta, Tokyo, Japan), which quantifies the optical density difference at 650 nm and 940 nm and produces a dimensionless index positively correlated with leaf chlorophyll concentration [34]. Canopy surface temperature (°C) was measured with a handheld infrared thermometer (±0.5 °C) directed at the upper canopy at a fixed distance and angle, providing an integrative indicator of stomatal conductance and plant water status under abiotic stress [35,36].

### 2.5. Statistical Analysis

All data were subjected to factorial analysis of variance (ANOVA) using Type III sums of squares to appropriately partition variance in an unbalanced design, as implemented in the Python (ver. 3.11.4) statsmodels library [37] via ordinary least squares (OLS) regression with full factorial model specification (G + S + M + G:S + G:M + S:M + G:S:M). The significance of main effects (genotype, salinity, melatonin) and all two- and three-way interactions was assessed at *p* ≤ 0.05, *p* ≤ 0.01, and *p* ≤ 0.001 probability levels. Prior to ANOVA, the assumptions of normality and homoscedasticity were evaluated using the Shapiro–Wilk test on model residuals and Levene’s test for equality of variances, respectively. Where deviations from normality were observed (*p* < 0.05), data were log-transformed prior to analysis to stabilise variance and improve symmetry of residuals. The factorial design generated a complete and balanced structure (13 × 3 × 4 × 3 = 468 observations) with equal replication across all treatment combinations; consequently, Type III sums of squares yield results equivalent to Type I sums under this design, with the choice of Type III ensuring robustness against any incidental imbalance from missing observations.

When a main effect or interaction was statistically significant, pairwise mean comparisons were performed using Tukey’s Honestly Significant Difference (HSD) test at the 5% significance level, implemented using the MultiComparison. For the salinity × melatonin interaction—the primary focus of this study—all 12 treatment combination means were separated using Tukey’s HSD, and homogeneous groups were identified by compact letter display. Data are presented as means ± standard error throughout.

## 3. Results

The factorial ANOVA revealed significant effects of the main factors and their interactions on most of the measured growth traits in forage pea genotypes (Table 1). Genotype (G), salinity (S), melatonin (M), and their interactions were evaluated for plant height, root length, number of nodes, leaf width, leaf length, internode distance, number of main branches, SPAD value, and canopy temperature.

### 3.1. Plant Height

**Figure 1:**
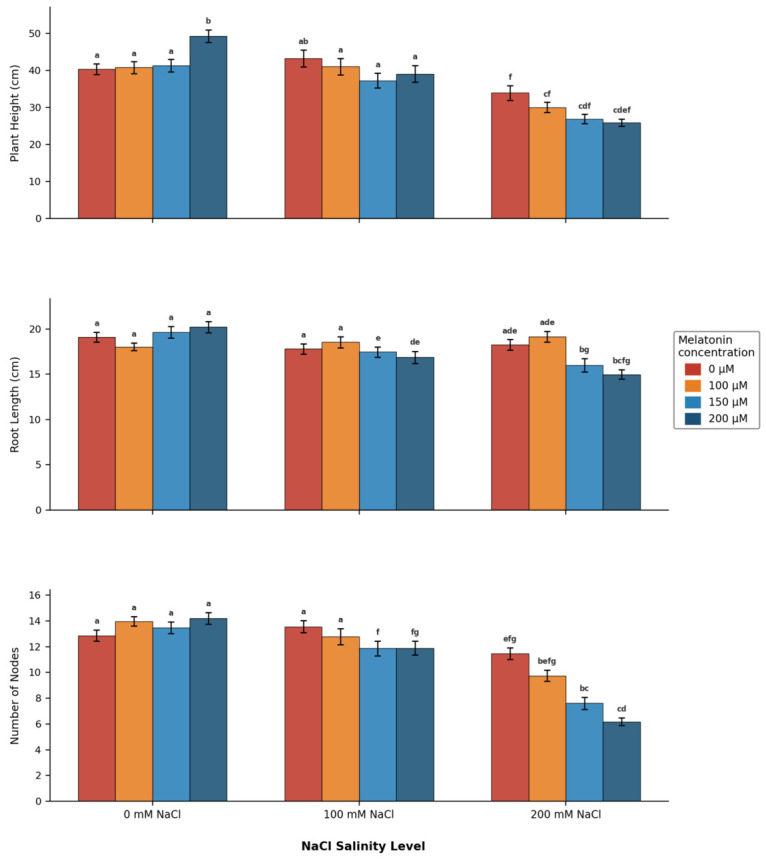
Effects of exogenous melatonin and NaCl salinity on plant height, root length, and number of nodes in forage pea (*Pisum sativum* L.) genotypes. Bars represent means ± standard error. Different letters above bars indicate significant differences among Salinity × Melatonin treatment combinations (Tukey’s HSD, *p* < 0.05).

Plant height was significantly affected by genotype (F_12,309_ = 4.84, *p* < 0.001), G × S (F_24,309_ = 3.64, *p* < 0.001), G × M (F_36,309_ = 2.15, *p* < 0.001) and the G × S × M three-way interaction (F_72,309_ = 2.50, *p* < 0.001), whereas the main effects of salinity (*p* = 0.055), melatonin (*p* = 0.234) and the S × M interaction (*p* > 0.05) were not significant—indicating a strongly genotype-conditioned response. The overall trend was a decline in plant height with increasing NaCl: from 42.97 ± 0.85 cm at 0 mM (group a) to 40.17 ± 1.10 cm at 100 mM (group a) and 29.20 ± 0.75 cm at 200 mM (group b). Among genotypes, Emirbey (54.29 ± 2.13 cm) and Kirazlí (50.36 ± 2.70 cm) attained the greatest heights, while Özkaynak (27.65 ± 1.57 cm) and Taşkent (29.24 ± 1.74 cm) were the shortest (Figure 1).

### 3.2. Root Length

Root length showed a significant S × M interaction (F_6309_ = 6.23, *p* < 0.001), a significant G × S × M three-way interaction (F_72,309_ = 2.23, *p* < 0.001) and a significant melatonin main effect (F_3309_ = 3.95, *p* < 0.01); main effects of genotype and salinity were not significant. Plants at 0 mM NaCl produced the longest roots (19.25 ± 0.28 cm, group a), followed by 100 mM (17.67 ± 0.30 cm, group b) and 200 mM NaCl (17.10 ± 0.33 cm). The S × M interaction revealed that under 200 mM NaCl, root length was 18.26 ± 0.58 cm without melatonin but decreased to 15.99 ± 0.72 cm and 14.98 ± 0.53 cm with 150 and 200 µM melatonin, respectively—an inhibitory response to high melatonin under severe salinity. Under non-saline conditions, root length remained consistent (18–20 cm) across all melatonin treatments (Figure 1).

### 3.3. Number of Nodes

Genotype (F_12,309_ = 4.03, *p* < 0.001), G × M (F_36,309_ = 2.11, *p* < 0.001), G × S × M (F_72,309_ = 2.15, *p* < 0.001) and G × S (F_24,309_ = 1.73, *p* < 0.05) significantly influenced node number. Salinity strongly reduced nodal development, with means declining from 13.63 ± 0.21 at 0 mM NaCl to 8.75 ± 0.26 at 200 mM NaCl. Under 200 mM NaCl, application of 150 and 200 µM melatonin produced the lowest node counts (7.62 and 6.18, respectively), indicating that elevated melatonin under severe salinity did not ameliorate—and may have compounded—the inhibition of nodal development (Figure 1).

### 3.4. Leaf Width

**Figure 2:**
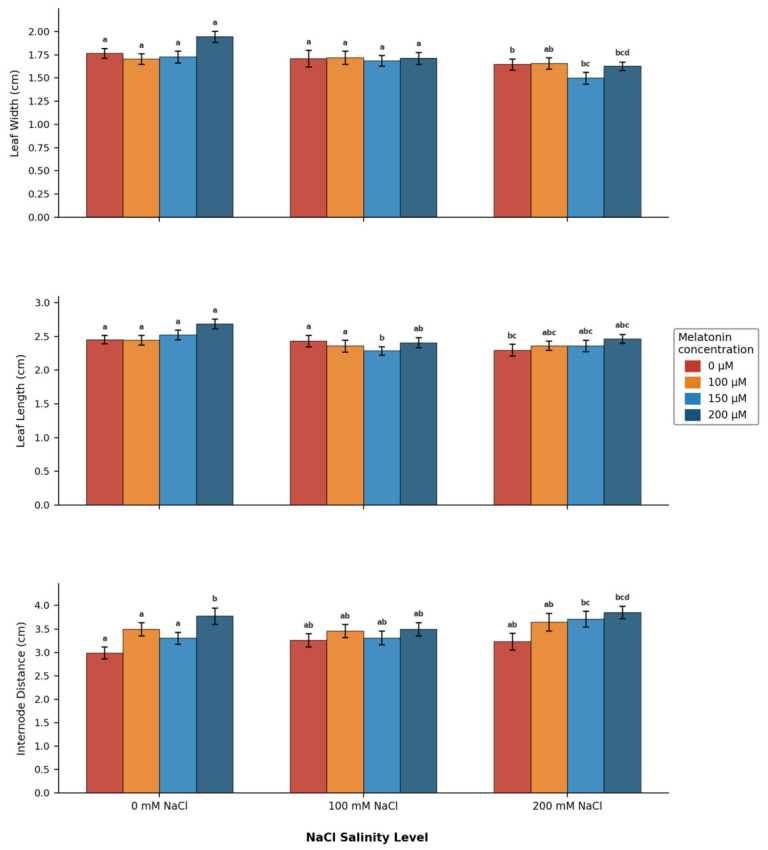
Effects of exogenous melatonin and NaCl salinity on leaf width, leaf length, and internode distance in forage pea genotypes. Bars represent means ± standard error. Different letters indicate significant differences (Tukey’s HSD, *p* < 0.05).

Leaf width was significantly affected by salinity (F_2309_ = 3.35, *p* < 0.05), melatonin (F_3309_ = 4.32, *p* < 0.01) and by the G × S, G × M, S × M and G × S × M interactions (all *p* < 0.01). Mean leaf width declined from 1.79 ± 0.03 cm at 0 mM NaCl to 1.61 ± 0.03 cm at 200 mM NaCl. Under 200 mM NaCl, 150 µM melatonin reduced leaf width to 1.50 ± 0.06 cm versus 1.65 ± 0.06 cm for the untreated control at the same salinity. Bölükbaşí exhibited the largest leaf width (2.21 ± 0.07 cm) and Kartalpínar the narrowest (1.39 ± 0.04 cm) (Figure 2).

### 3.5. Leaf Length

Melatonin significantly affected leaf length (F_3309_ = 2.90, *p* < 0.05), as did the G × S (*p* < 0.05) and G × S × M (*p* < 0.01) interactions. Leaf length declined from 2.53 ± 0.04 cm at 0 mM NaCl to 2.37 ± 0.04 cm at 100 mM NaCl, with no further significant decrease at 200 mM. Bölükbaşí produced the longest leaves (3.04 ± 0.06 cm) and Özkaynak the shortest (2.13 ± 0.08 cm) (Figure 2).

### 3.6. Internode Distance

Internode distance was significantly influenced by genotype (*p* < 0.01), melatonin (*p* < 0.05), and the G × S, G × M and G × S × M interactions. Internode distance tended to increase under salt stress and with higher melatonin levels, reaching a maximum of 3.86 ± 0.13 cm under 200 mM NaCl with 200 µM melatonin compared with 2.99 ± 0.13 cm at 0 mM NaCl without melatonin. Emirbey (4.56 cm) and Kirazlí (4.39 cm) showed the greatest internode distances (Figure 2).

### 3.7. Number of Main Branches

**Figure 3:**
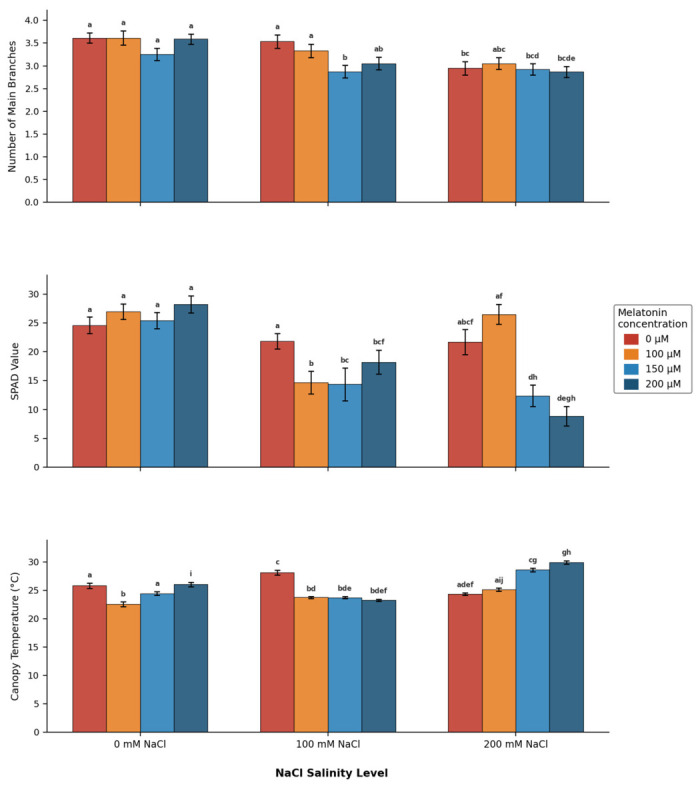
Effects of exogenous melatonin and NaCl salinity on number of main branches, SPAD value (relative chlorophyll content), and canopy temperature in forage pea genotypes. Bars represent means ± standard error. Different letters indicate significant differences (Tukey’s HSD, *p* < 0.05).

Genotype (F_12,309_ = 3.25, *p* < 0.001), G × M (*p* < 0.05) and G × S × M (*p* < 0.05) significantly affected the number of main branches; the S × M interaction was not significant. Branch number decreased progressively with salinity: 3.52 ± 0.07 at 0 mM (group a), 3.20 ± 0.07 at 100 mM (group b) and 2.95 ± 0.06 at 200 mM (group c). Application of 150 µM melatonin under 100 mM NaCl produced the lowest branch number among the 100 mM treatments (2.87 ± 0.14). Bölükbaşí had the highest mean (3.72 ± 0.09) and Ürünlü the lowest (1.88 ± 0.16) (Figure 3).

### 3.8. SPAD Value (Chlorophyll Content)

SPAD values were significantly influenced by genotype (*p* < 0.05), G × S (*p* < 0.001), S × M (*p* < 0.01) and G × S × M (*p* < 0.001). Mean SPAD declined from 26.30 ± 0.70 at 0 mM NaCl to 17.25 ± 1.08 at 100 mM NaCl. The S × M interaction revealed a complex response: under non-saline conditions, melatonin application maintained or slightly elevated SPAD (highest at 200 µM, 28.24 ± 1.46). Under 100 mM NaCl, all melatonin treatments reduced SPAD relative to the untreated 0 mM control, with 150 µM producing the lowest value (14.36 ± 2.82). Under 200 mM NaCl, the untreated control retained SPAD at 21.66 ± 2.17, whereas 100 µM melatonin partially restored values to 26.50 ± 1.72; 200 µM melatonin at the same salinity produced the lowest value recorded (8.86 ± 1.70). Özkaynak had the highest mean SPAD (29.50 ± 2.83) and Paslí the lowest (15.44 ± 1.90) (Figure 3).

### 3.9. Canopy Temperature

Canopy temperature was the trait most affected by all factors, with significant main effects of genotype (F_12,309_ = 14.79, *p* < 0.001), salinity (*p* < 0.05) and melatonin (F_3309_ = 34.78, *p* < 0.001), as well as all two- and three-way interactions (all *p* < 0.001). Mean canopy temperature was elevated at 200 mM NaCl (26.98 ± 0.23 °C, group b) relative to 0 mM (24.70 ± 0.23 °C, group a) and 100 mM (24.71 ± 0.20 °C). Across melatonin levels, 100 µM produced the lowest mean canopy temperature (23.79 ± 0.21 °C). The S × M interaction was particularly striking: under 100 mM NaCl, 200 µM melatonin lowered canopy temperature from 28.12 ± 0.40 °C to 23.24 ± 0.17 °C, whereas under 200 mM NaCl the inverse pattern emerged, with canopy temperature rising from 24.34 ± 0.23 °C without melatonin to 29.91 ± 0.28 °C with 200 µM melatonin (Figure 3).

**Figure 4:**
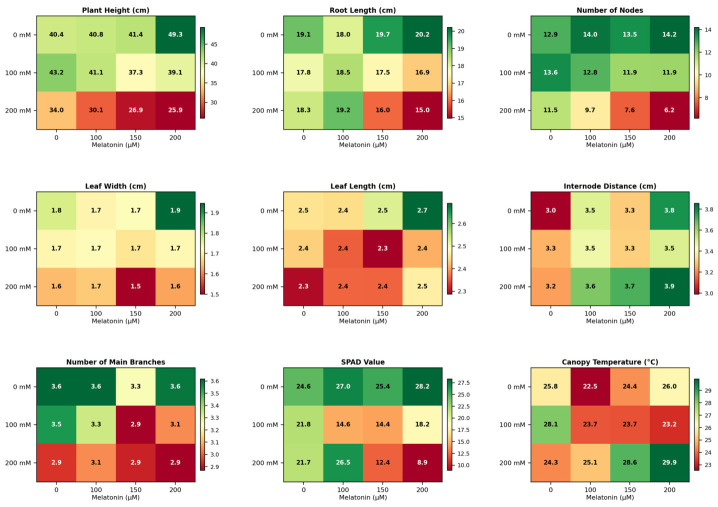
Heatmap of mean trait values across all Salinity × Melatonin treatment combinations. Green color indicates higher values; red indicates lower values. Cell values are treatment means.

Genotypic responses across the three salinity levels for plant height, SPAD value and canopy temperature are summarised in Figure 4, illustrating that Emirbey and Kirazlí maintained the greatest vegetative growth across all salinity levels, while Özkaynak preserved the highest SPAD under stress.

Figure 4 provides an integrative summary of mean trait values across all salinity × melatonin treatment combinations.

## 4. Discussion

The present study examined how exogenous melatonin application interacts with NaCl-induced salinity stress to shape the vegetative growth of 13 forage pea (*Pisum sativum* L.) genotypes under greenhouse conditions. Salinity stress exerted broadly negative effects on vegetative traits, while the response to melatonin was strongly dependent on both genotype and concentration, with no single treatment providing consistent benefits across all traits and salinity levels.

### 4.1. Effects of Salinity on Forage Pea Growth

Increasing NaCl progressively reduced plant height, node number, leaf dimensions and number of main branches, while elevating canopy temperature. These responses align with the established understanding that high NaCl concentrations induce osmotic stress, ionic toxicity—primarily through Na^+^ and Cl^−^ accumulation—and nutritional imbalances that collectively impair cell division, elongation and differentiation in legumes [2,4]. The ~32% decline in plant height observed at 200 mM NaCl mirrors the severe growth suppression reported at comparable salinities in garden pea [15], and the parallel reduction in biomass, relative water content and seedling vigour previously documented by Gaber et al. [14] in field pea, and is consistent with the patterns reported in cowpea and Italian zucchini under comparable salt stress [38,39].

The marked decline in SPAD values under NaCl stress (from 26.30 at 0 mM to 17.25 at 100 mM NaCl) reflects the interplay of multiple disrupting mechanisms. Salt-induced oxidative stress generates ROS that directly oxidise chlorophyll molecules and damage the photosynthetic electron transport chain, with thylakoid membranes being particularly vulnerable [2,20]. Concurrently, ionic imbalance impairs chlorophyll biosynthesis: Na^+^ competitively displaces Mg^2+^ from the chloroplast envelope and disrupts Mg^2+^-chelatase, the first committed enzyme of the chlorophyll biosynthetic pathway [40]. Hormonal imbalance, particularly elevated ABA and reduced cytokinin levels under salt stress, further accelerates chlorophyll degradation through senescence-associated pathways [41]. The fact that 100 µM melatonin under 200 mM NaCl restored SPAD to 26.50 (from 21.66 in the untreated saline control) is therefore likely attributable to melatonin’s dual ROS-scavenging and senescence-delaying actions, which have been documented for cucumber and other crops under salinity [42].

The concurrent elevation of canopy temperature at 200 mM NaCl is consistent with reduced transpirational cooling under stomatal closure, a canonical adaptive response that limits water loss at the expense of CO_2_ fixation [6]. The increase, rather than decrease, in internode distance under salinity is intriguing and may reflect a resource-reallocation strategy in which limited carbohydrate supplies are channelled toward stem elongation at the expense of lateral organ production, possibly mediated by salt-induced redistribution of auxin [19].

### 4.2. Effects of Exogenous Melatonin Under Salt Stress

Although the main effects of melatonin were generally non-significant for shoot growth traits such as plant height and number of main branches, significant effects were observed for root length, leaf dimensions, internode distance and canopy temperature. The highly significant G × S × M three-way interactions across nearly all traits indicate that the response to melatonin is strongly modulated by genotypic background and salinity regime. This pattern aligns with the meta-analytic findings of Qi et al. [21], who reported that 50–200 µM is the broadly effective range for melatonin under salt stress but emphasised that efficacy depends critically on timing, dose, species and stress severity. In our study, 200 µM melatonin enhanced plant height under non-saline conditions (49.34 cm vs. 40.38 cm in untreated controls), consistent with melatonin’s well-described auxin-like promotion of cell elongation [7]. Under 200 mM NaCl, by contrast, melatonin application failed to restore plant height, root length and node number, and in some instances reduced these traits—paralleling earlier observations in rice where 100 µM was optimal while higher doses became neutral or inhibitory [18].

The SPAD response to melatonin was particularly complex. Under non-saline conditions, all melatonin doses maintained or slightly elevated chlorophyll content. Under 100 mM NaCl, however, melatonin reduced SPAD values—a finding that contrasts with reports in horsegram, in which 100–150 µM melatonin preserved chlorophyll and carotenoid content under salinity [30]. This discrepancy may reflect species-specific sensitivities of *P. sativum* to exogenous melatonin, or interactions between melatonin-mediated tryptophan metabolism and chlorophyll degradation pathways described in *Phaseolus vulgaris* [19]. Under severe stress (200 mM NaCl), 100 µM melatonin partially restored SPAD to 26.50, whereas 200 µM produced the lowest recorded value (8.86), pointing to a paradoxical exacerbation of oxidative damage to the photosynthetic apparatus at supra-optimal doses.

The reduction in canopy temperature elicited by 100 µM melatonin under 100 mM NaCl (from 28.12 °C to 23.74 °C) is of particular practical interest. This response suggests that melatonin at moderate concentrations promotes stomatal conductance and transpirational cooling, possibly through modulation of ABA signalling. A comparable mechanism has been proposed for maize, in which melatonin elevated SOD, CAT and POD activities and reduced Na^+^ accumulation, improving leaf water status and stomatal function under salinity [43]. The reversal of this pattern under 200 mM NaCl—where 150 and 200 µM melatonin instead raised canopy temperature to 28–30 °C—suggests that at severe salinity levels, melatonin-induced metabolic adjustments may be insufficient to compensate for root dysfunction driven by high ionic toxicity.

### 4.3. Genotypic Variation in Salt Tolerance

Significant genotypic main effects were detected for plant height, node number, SPAD, canopy temperature, internode distance and main branches. Emirbey and Kirazlí consistently produced the greatest vegetative growth across all treatments, while Özkaynak and Taşkent were among the least productive. The strongly significant G × S and G × M interactions confirm that genotypes differ substantially in their plasticity toward salinity and melatonin—an observation consistent with the documented genetic diversity for salt tolerance in *Pisum sativum* [23,24]. Maurya et al. [30] similarly reported that tolerant horsegram genotypes responded more favourably to melatonin under salt stress than sensitive ones, underscoring the need to account for genotypic background when applying exogenous bioregulators. The significant three-way G × S × M interaction observed here for all nine traits has direct practical consequences: a single melatonin recommendation is unlikely to be transferable across all genotypes or all salinity regimes, and the identification of effective genotype–dose combinations under specific salinity conditions necessarily requires the type of multifactorial screening implemented in the present study.

The pervasive three-way G × S × M interactions detected for nearly all traits warrant deeper mechanistic interpretation. These interactions reveal that the physiological response to exogenous melatonin is not merely a function of dose, nor of salinity intensity alone, but emerges from the interplay between genotype-specific antioxidant capacity, ion-exclusion machinery (Na^+^/H^+^ antiporters, SOS pathway), and tissue water relations. Emirbey and Kirazlí sustained vegetative growth even at 200 mM NaCl with 100 µM melatonin, suggesting effective ionic regulation and osmotic adjustment, whereas Özkaynak retained chlorophyll despite limited stature, implying preferential allocation of melatonin-mediated protection to the photosynthetic apparatus rather than to elongation growth. Such divergent adaptive strategies among genotypes mirror observations of varietal heterogeneity in tomato, cowpea, and Italian zucchini exposed to salinity [10,38,39], reinforcing the principle that biostimulant efficacy is contingent on the genetic background’s adaptive plasticity and that genotype–biostimulant matching is essential for translation into practice.

### 4.4. Optimum Melatonin Concentration and Practical Implications

Across the measured traits, 100 µM melatonin was associated with the lowest canopy temperature under moderate salt stress, maintenance or improvement of root length under non-saline conditions, and relatively stable SPAD values compared with 150 and 200 µM under several salinity scenarios. This response is broadly consistent with the 50–200 µM optimal range identified by Qi et al. [21] and with specific improvements observed at 100 µM in salt-stressed legumes [29,44]. Abdelhafez et al. [29] reported that co-application of ACC deaminase-producing rhizobia and melatonin improved ion homeostasis and growth parameters under salinity in *Phaseolus vulgaris*, suggesting that synergistic combinations of melatonin with microbial biostimulants warrant further exploration in forage pea. By contrast, 150 and 200 µM melatonin were often associated with the lowest trait values under 200 mM NaCl, suggesting a dose-dependent interaction above a critical threshold. This phenomenon—where high exogenous melatonin becomes inhibitory under extreme stress—has been documented in sorghum [45] and rice [18] and likely reflects disruption of endogenous hormone balance or interference with tryptophan metabolism at supra-optimal melatonin concentrations.

It is critical to acknowledge that the present study did not directly measure dry biomass or forage yield—a limitation that constrains direct claims about agronomic productivity. The morphological indices reported (height, branching, leaf dimensions) and chlorophyll/canopy indices (SPAD, canopy temperature) are widely accepted proxies for biomass accumulation and photosynthetic performance, but they cannot substitute for direct gravimetric yield data. Within these constraints, the data support a foliar application of 100 µM melatonin for forage pea cultivated on moderately saline soils (≤100 mM NaCl equivalent), particularly when paired with relatively tolerant genotypes such as Emirbey and Kirazlí. The preservation of leaf dimensions and SPAD under moderate salinity at 100 µM is especially relevant from a forage quality perspective, as these traits underpin photosynthetic efficiency and ultimately biomass and nutritive value in forage legumes. Direct confirmation of agronomic value, however, requires multi-season field trials integrating gravimetric yield, forage quality (crude protein, neutral detergent fibre, in vitro digestibility), and economic analysis.

### 4.5. Mechanistic Insights: Cytoskeleton, Osmoprotectants, and Metabolic Responses

The paradoxical toxic effect of high melatonin doses under severe salinity (150–200 µM at 200 mM NaCl) suggests a complex interference with normal cellular adaptation mechanisms. Salt stress triggers specific patterns of osmotic adjustment and cell wall/cytoskeletal reorganization; excessive melatonin may disrupt these critical responses. The recent literature has identified the reorganization of cortical microtubules and interphase microtubule arrays as a key adaptive mechanism in root cells during osmotic and salt stress [23,24]. Microtubule reorientation is essential for maintaining cell shape and enabling polarised growth in conditions of osmotic imbalance. Exogenous melatonin, at supra-optimal concentrations, may interfere with the normal signalling cascades—possibly including calcium-dependent pathways—that coordinate microtubule reorganization, thereby compromising the cytoskeletal adaptation essential for growth under severe stress.

Osmotic adjustment through the accumulation of compatible solutes (osmoprotectants) is a central mechanism of salt tolerance. Primary osmoprotectants such as proline, betaine, and free sugars maintain cell turgor while avoiding ion accumulation; secondary osmoprotectants such as phenolic compounds and anthocyanins enhance antioxidant defence [25,26]. Melatonin is recognised as a signalling molecule that modulates gene expression of key enzymes in osmoprotectant synthesis, including Δ1-pyrroline-5-carboxylate synthetase (P5CS, the first committed step of proline synthesis) and trehalose synthase. At moderate doses (100 µM), melatonin likely promotes the upregulation of these genes, facilitating osmoprotectant accumulation consistent with the improved growth observed in our study. At supra-optimal doses (150–200 µM), melatonin may overwhelm the cellular capacity for osmolyte synthesis or trigger pro-senescence signalling pathways—including programmed cell death (PCD) responses—that have been shown to be activated by excessive oxidative signalling and disrupted auxin homeostasis under combined stress [27,28]. The concentration-dependent switch from protective to inhibitory effects likely reflects a critical threshold in the balance between melatonin’s antioxidant capacity and its signalling roles in growth regulation.

Why was metabolite quantification not undertaken in the present study, despite publication in a journal devoted to metabolites? The answer reflects both pragmatic constraints and deliberate research design. A comprehensive metabolomic survey—untargeted LC-MS/MS or GC-MS profiling of primary and secondary metabolites across 13 genotypes × 3 salinity levels × 4 melatonin doses × 3 replications (468 samples)—would entail substantial instrumentation cost, specialist expertise in bioinformatics, and extended analysis timeframes incompatible with the timely dissemination of the plant phenotypic discoveries reported here. Future studies should adopt a targeted approach, focusing on a smaller subset of contrasting genotypes (e.g., high-responders Emirbey and Kirazlí vs. low-responders Özkaynak and Taşkent) and measuring key metabolites directly implicated in the proposed mechanisms: proline, betaine, and total soluble sugars (as primary osmoprotectants); malondialdehyde and protein carbonyls (as markers of oxidative damage); and cis-zeatin, gibberellins, and abscisic acid (as hormonal correlates of the growth and stress responses). Such metabolomic profiling would directly test whether melatonin-induced changes in growth are accompanied by the predicted shifts in osmoprotectant accumulation and metabolic reorientation.

Future studies should extend these findings to field conditions across multiple growing seasons to account for soil heterogeneity, climatic variation and crop developmental stages beyond the vegetative phase examined here. Biochemical and molecular work—quantifying reactive oxygen species, antioxidant enzyme activities and expression of stress-responsive genes such as *SOS1*, *NHX* and *DREB*—would help illuminate the mechanisms underlying the observed genotypic differences in melatonin responsiveness. Complementary evaluation of yield components (pod number, seed weight, dry-matter yield) and fodder quality parameters (crude protein, neutral detergent fibre) under saline conditions and with melatonin treatment would provide the agronomically complete picture required to translate these greenhouse findings into practical recommendations for forage pea production in salt-affected agroecosystems.

## 5. Limitations

This study has several important limitations that should be acknowledged. First, the experiment was conducted in a controlled greenhouse environment with artificial and supplemental natural lighting, which does not fully replicate field conditions characterised by variable light quality, intensity, and photoperiod. Second, plant samples were harvested only once, at 15 days after treatment initiation, providing a snapshot of the vegetative phase but not capturing longer-term biomass accumulation, reproductive development, or yield components essential for evaluating agronomic viability. Third, no photographic documentation of visual plant symptoms or wilting was collected, which would have strengthened the qualitative assessment of stress severity and treatment effects. Fourth, the scope of metabolite analysis was limited to morphological and physiological indices (leaf area, SPAD, canopy temperature); direct quantification of primary metabolites (soluble sugars, proline, betaine) and secondary metabolites (phenolics, anthocyanins) or gene expression of key enzymes would have provided mechanistic depth. Fifth, thermal imaging was not employed to confirm canopy temperature measurements, although the handheld infrared thermometer used provides adequate precision for the differences detected.

Most critically, no direct measurement of dry biomass, forage yield, or quality parameters (crude protein, neutral detergent fibre, in vitro digestibility) was performed. The morphological and physiological indices examined here—plant height, branching, leaf dimensions, SPAD, canopy temperature—are widely accepted proxies for biomass accumulation and photosynthetic performance under controlled conditions, but they cannot substitute for direct gravimetric yield data under field conditions. Consequently, while the present study identifies promising genotype–dose combinations and articulates a mechanistic framework for melatonin–salinity interactions, claims of agronomic effectiveness require validation through multi-season field productivity trials. Such trials should quantify forage yield, quality parameters, and the economic return of melatonin application relative to its cost, before recommendations can be made for widespread deployment in commercial forage production on salt-affected soils.

## 6. Conclusions

Exogenous melatonin can partially counteract NaCl-induced inhibition of vegetative growth in forage pea, but its efficacy is conditioned by both concentration and genotype. Across 13 genotypes, 100 µM melatonin emerged as the optimal dose, lowering canopy temperature from 28.1 °C to 23.7 °C under 100 mM NaCl and restoring SPAD from 21.7 to 26.5 under 200 mM NaCl. Supra-optimal concentrations (150–200 µM) under severe salinity produced a paradoxical phytotoxic response, depressing SPAD to 8.9 and shortening roots, indicating a clear concentration-dependent threshold. Genotype was a major source of variation: Emirbey and Kirazlí sustained the highest vegetative growth across treatments, whereas Özkaynak retained the greatest chlorophyll content. The significant three-way genotype × salinity × melatonin interaction confirmed that no single melatonin dose is universally optimal. Coupling a 100 µM foliar application with targeted genotype selection therefore offers a tractable, low-input strategy for improving forage pea performance on moderately saline agricultural soils. It must be emphasised, however, that the findings reported here pertain to vegetative-phase morphological and physiological responses; agronomic effectiveness cannot be claimed without direct measurement of biomass, yield, and forage quality under field conditions, which represents the priority next step. Future work should integrate metabolomic profiling, field productivity trials, and mechanistic studies of cytoskeletal and osmoprotectant responses to fully elucidate melatonin’s role in salt stress mitigation and to enable confident agronomic deployment.

## Figures and Tables

**Table 1:** Factorial ANOVA F-values and significance levels for nine growth traits across genotype (G), salinity (S) and melatonin (M) main effects and their interactions. ns = not significant; * *p* < 0.05; ** *p* < 0.01; *** *p* < 0.001.

Trait	Genotype (G)	Salinity (S)	Melatonin (M)	G × S	G × M	S × M	G × S × M
Plant Height	4.84 ***	2.93 ns	1.43 ns	3.64 ***	2.15 ***	1.00 ns	2.50 ***
Root Length	1.13 ns	1.37 ns	3.95 **	1.51 ns	1.35 ns	6.23 ***	2.23 ***
No. of Nodes	4.03 ***	1.04 ns	0.53 ns	1.73 *	2.11 ***	1.94 ns	2.15 ***
Leaf Width	1.60 ns	3.35 *	4.32 **	2.75 ***	1.90 **	2.76 *	1.68 **
Leaf Length	1.09 ns	0.23 ns	2.90 *	1.60 *	1.22 ns	0.67 ns	1.51 **
Internode Dist.	2.27 **	1.89 ns	3.75 *	1.90 **	1.46 *	1.16 ns	1.72 ***
Main Branches	3.25 ***	0.00 ns	0.78 ns	1.50 ns	1.66 *	0.63 ns	1.46 *
SPAD	2.18 *	1.86 ns	1.48 ns	2.49 ***	1.17 ns	2.87 **	2.69 ***
Canopy Temp.	14.79 ***	4.50 *	34.78 ***	10.19 ***	9.79 ***	17.14 ***	5.67 ***

## Data Availability

The data that support the findings of this study are available from the corresponding author upon reasonable request.

## References

[1] FAO (2024). Global Status of Salt-Affected Soils.

[2] Singh N., Maurya V., Gupta K., Sharma I., Sharma A., Kumar R. (2025). Salt stress and its eco-friendly management using biostimulants in grain legumes: A review. Discov. Agric..

[3] Cüllü M.A., Aydemir S., Qadir M., Almaca A., Öztürkmen A.R., Bilgiç A., Ağca N. (2010). Implication of groundwater fluctuation on the seasonal salt dynamic in the Harran Plain, south-eastern Turkey. Irrig. Drain..

[4] Ehtaiwwesh A.F., Emsahel M.J. (2020). Impact of salinity stress on germination and growth of pea (*Pisum sativum* L.) plants. Al-Mukhtar J. Sci..

[5] Munns R., Tester M. (2008). Mechanisms of salinity tolerance. Annu. Rev. Plant Biol..

[6] Zhan H., Weining S., Li S., Zhang T., Tong W., Du X., Nie X., Wang X. (2019). Melatonin: A small molecule but important for salt stress tolerance in plants. Int. J. Mol. Sci..

[7] Zhao C., Liu J., Li J., Li L., Chen M., Zhu T. (2019). The role of melatonin in salt stress responses. Int. J. Mol. Sci..

[8] Ilahi H., Zampieri E., Sbrana C., Brescia F., Giovannini L., Mahmoudi R., Gohari G., El Idrissi M.M., Alfeddy M.N., Schillaci M. (2024). Impact of two Erwinia sp. on the response of diverse *Pisum sativum* genotypes under salt stress. Physiol. Mol. Biol. Plants.

[9] Alves T.R.C., Cavalcante L.F., Lima G.S., Soares L.A.A., Bonifácio B.F., Andrade M.E.O., Souza J.A.A., Sales R.S., Lima R.F., Souza A.F.M. (2022). Production and physiological quality of seeds of mini watermelon grown in substrates with saline nutrient solution prepared with reject brine. Plants.

[10] Loiola A.T., Carvalho A.F.U., Cavalcante A.C.R., Alencar N.M.M., Sousa N.S., Pessoa R.S., Praxedes S.C., Pereira M.G.S., Carvalho M.S.L., Bezerra A.M.E. (2022). Phenology and production of traditional seeds of cowpea irrigated with saline water. Rev. Ciência Agronômica.

[11] Thavarajah D., Powers S.E. (2019). Checking agriculture’s pulse: Field pea (*Pisum sativum* L.), sustainability, and phosphorus use efficiency. Front. Plant Sci..

[12] Piltz J., Rodham C. (2022). Effect of sowing rate and maturity on the yield and nutritive value of triticale–field pea forage crops. Sustainability.

[13] Thavarajah D., Shipe E., Kay J., McGee R., Boyles R.E., Powers S.E., Kumar S., Lawrence T.J. (2022). Organic dry pea (*Pisum sativum* L.) biofortification for better human health. PLoS ONE.

[14] Gaber A., Hossain A., Alsuhaibani A.M., Sarker K.K., Brestič M., Skalicý M., Mia M.A.B., Quddus M.A., Khan M.A.H., Rahman M. (2022). Salinity-induced physiological changes in pea (*Pisum sativum* L.): Germination rate, biomass accumulation, relative water content, seedling vigor and salt tolerance index. Plants.

[15] Grozeva S., Kalapchieva S., Tringovska I. (2023). In vitro screening for salinity tolerance in garden pea (*Pisum sativum* L.). Horticulturae.

[16] Reiter R.J., Tan D.X., Burkhardt S., Manchester L.C. (2001). Melatonin in plants. Nutr. Rev..

[17] Jin B., Mostafa S., Zeng W., Lu Z. (2022). Melatonin-mediated abiotic stress tolerance in plants. Front. Plant Sci..

[18] Khan Z., Jan R., Asif S., Farooq M., Jang Y.H., Kim E.G., Kim N., Kim K.M. (2024). Exogenous melatonin induces salt and drought stress tolerance in rice by promoting plant growth and defense system. Sci. Rep..

[19] Yang X., Liu D., Liu C., Li M., Yan Z., Zhang Y., Feng G. (2024). Possible melatonin-induced salt stress tolerance pathway in *Phaseolus vulgaris* L. using transcriptomic and metabolomic analyses. BMC Plant Biol..

[20] Karumannil S., Khan T.A., Kappachery S., Gururani M. (2023). Impact of exogenous melatonin application on photosynthetic machinery under abiotic stress conditions. Plants.

[21] Qi B., Yan F., Li G., Zhang G., Zhao H., Zhang L., Wu L., Fan S., Ding Y., Niu Y.P. (2022). Basic cognition of melatonin regulation of plant growth under salt stress: A meta-analysis. Antioxidants.

[22] Ahmad R., Alsahli A., Alansi S., Altaf M.A. (2023). Exogenous melatonin confers drought stress tolerance by promoting plant growth, photosynthetic efficiency and antioxidant defense system of pea (*Pisum sativum* L.). Sci. Hortic..

[23] Lazareva E.M., Baranova E.N., Smirnova E.A. (2017). Reorganization of interphase microtubules in root cells of *Medicago sativa* L. during acclimation to osmotic and salt stress. Cell Tissue Biol..

[24] Mehta D., Vyas S. (2023). Comparative bio-accumulation of osmoprotectants in saline stress-tolerating plants: A review. Plant Stress.

[25] Ur Rahman S., Basit A., Ara N., Ullah I., Rehman A.U. (2021). Morpho-physiological responses of tomato genotypes under saline conditions. Gesunde Pflanz..

[26] Fedoreyeva L.I., Lazareva E.M., Shelepova O.V., Baranova E.N., Kononenko N.V. (2022). Salt-induced autophagy and programmed cell death in wheat. Agronomy.

[27] Kononenko N., Baranova E., Dilovarova T., Akanov E., Fedoreyeva L. (2020). Signalling molecules and cell death in salt stress response. Agriculture.

[28] Mittler R. (2002). Oxidative stress, antioxidants and stress tolerance. Trends Plant Sci..

[29] Abdelhafez A.A., Dadkhodaie A., El-Ghareeb D., Alinia M., Bassouny M., Sepehri M., Poczai P., Amjad S., Kazemeini S.A., Mahjenabadi V.A.J. (2022). Co-application of ACC deaminase-producing rhizobial bacteria and melatonin improves salt tolerance in common bean (*Phaseolus vulgaris* L.) through ion homeostasis. Sci. Rep..

[30] Maurya V., Singh N., Sharma A., Kumar R. (2026). Ameliorative Effect of Melatonin on Morphological and Biochemical Parameters of Salt-Tolerant and Sensitive Genotypes of *Macrotyloma uniflorum* (L.) Verdc. Biotechnol. Appl. Biochem..

[31] Arnao M.B., Hernández-Ruiz J. (2018). Melatonin and its relationship to plant hormones. Ann. Bot..

[32] Weeda S., Zhang N., Zhao X., Ndip G., Guo Y., Buck G.A., Fu C., Ren S. (2014). Arabidopsis transcriptome analysis reveals key roles of melatonin in plant defense against UV radiation. J. Pineal Res..

[33] Özel S.D., Gökkuş A., Alatürk F. (2016). Farklı Sulama Seviyelerinin Macar Fiği (*Vicia pannonica* Crantz.) Ve Yem Bezelyesinin (*Pisum arvense* L.) Gelişimine Etkileri. Alınteri.

[34] Shah S.H., Houborg R., McCabe M.F. (2017). Response of chlorophyll, carotenoid and SPAD-502 measurement to salinity and nutrient stress in wheat (*Triticum aestivum* L.). Agronomy.

[35] Fuchs M. (1990). Infrared measurement of canopy temperature and detection of plant water stress. Theor. Appl. Climatol..

[36] Mayanja I.K., Diepenbrock C.H., Vadez V., Lei T., Bailey B.N. (2024). Practical considerations and limitations of using leaf and canopy temperature measurements as a stomatal conductance proxy: Sensitivity across environmental conditions, scale, and sample size. Plant Phenomics.

[37] Seabold S., Perktold J. Statsmodels: Econometric and statistical modeling with Python. Proceedings of the 9th Python in Science Conference.

[38] Praxedes S.S.C., Ferreira T.B., Carvalho A.J., Lima V.F., Marques E.C., Lacerda C.F., Carvalho F.E.L. (2022). Photosynthetic responses, growth, production, and tolerance of traditional varieties of cowpea under salt stress. Plants.

[39] Fernandes C.S., Soares L.A.A., Lima G.S., Gheyi H.R., Saboya L.M.F., Ferreira J.T.A. (2022). Ionic homeostasis, biochemical components and yield of Italian zucchini under nitrogen forms and salt stress. Braz. J. Biol..

[40] Tanaka R., Tanaka A. (2007). Tetrapyrrole biosynthesis in higher plants. Annu. Rev. Plant Biol..

[41] Argueso C.T., Ferreira F.J., Kieber J.J. (2009). Environmental perception avenues: The interaction of cytokinin and environmental response pathways. Plant Cell Environ..

[42] Zhang H.J., Zhang N., Yang R.C., Wang L., Sun Q.Q., Li D.B., Cao Y.Y., Weeda S., Zhao B., Ren S. (2014). Melatonin promotes seed germination under high salinity by regulating antioxidant systems, ABA and GA4 interaction in cucumber (*Cucumis sativus* L.). J. Pineal Res..

[43] Li W.Q., Li J., Bi S., Jin J., Fan Z.L., Shang Z.L., Zhang Y., Wang Y.J. (2025). Melatonin enhances maize germination, growth, and salt tolerance by regulating reactive oxygen species accumulation and antioxidant systems. Plants.

[44] Jalili S., Ehsanpour A., Morteza Javadirad S. (2023). Melatonin improves salt tolerance of in vitro root culture of alfalfa (*Medicago sativa* L.). Biologia.

[45] Helal N.M., Saudy H.S., Hamada M.M.A., El-Yazied A.A., El-Gawad H.G.A., Mukherjee S., Al-Qahtani S.M., Al-Harbi N.A., El-Sayed S.M., Ibrahim M.F.M. (2024). Potentiality of melatonin for reinforcing salinity tolerance in sorghum seedlings via boosting photosynthetic pigments, ionic and osmotic homeostasis and reducing the carbonyl/oxidative stress markers. J. Soil Sci. Plant Nutr..

